# Recovery of *Saccharomyces cerevisiae* CNCM I-3856 in Vaginal Samples of Healthy Women after Oral Administration

**DOI:** 10.3390/nu12082211

**Published:** 2020-07-24

**Authors:** Amelie Decherf, Elodie Dehay, Mickaël Boyer, Mathieu Clément-Ziza, Bertrand Rodriguez, Sophie Legrain-Raspaud

**Affiliations:** 1 Research and Applications Department, Gnosis by Lesaffre, Lesaffre Group, 59700 Marcq-en-Baroeul, France; e.dehay@gnosis.lesaffre.com (E.D.); b.rodriguez@gnosis.lesaffre.com (B.R.); s.legrain@gnosis.lesaffre.com (S.L.-R.); 2 Microbiology Laboratory, Research and Development Department, Lesaffre International, Lesaffre Group, 59700 Marcq-en-Baroeul, France; m.boyer@lesaffre.com; 3 Data Science and Bioinformatics Laboratory, Research and Development Department, Lesaffre International, Lesaffre Group, 59700 Marcq-en-Baroeul, France; m.clementziza@lesaffre.com

**Keywords:** probiotic, *Saccharomyces cerevisiae*, vaginal infection, vaginal health, microbiota, prevention, yeasts

## Abstract

Bacterial vaginosis and vulvovaginal candidiasis are common causes of impaired health and quality of life for women. Although antimicrobial agents remain the main strategy for the treatment of vaginal infections, their repeated use involves high rates of resistance and recurrence. Alternative approaches such as probiotics are studied. *Saccharomyces cerevisiae* CNCM I-3856 already demonstrated beneficial effects in experimental models of vaginal infections. This randomized, double-blind, placebo-controlled clinical study was performed to evaluate the recovery of *S. cerevisiae* CNCM I-3856 in vaginal samples in healthy women after oral consumption. Sixty healthy women were randomized to receive a daily dose of *S. cerevisiae* CNCM I-3856 or a placebo for 4 weeks. Subcultures and quantitative polymerase chain reaction (qPCR) were used to detect the strain in vaginal and stool samples. A safety assessment was carried out throughout the study. Fifty-seven women completed the study. Over the 4-week supplementation phase, *S. cerevisiae* CNCM I-3856 has been detected in the vaginal samples of 21% of women (*n* = 4/19) in the 500 mg Probiotic group and 16% of women (*n* = 3/19) in the 1000 mg Probiotic group. The strain was detected in the faeces of 90% of women consuming the probiotic. This is the first clinical study demonstrating the migration of yeast from intestine to vagina where it may exert its benefits.

## 1. Introduction

The vaginal microbiota is a dynamic microbial community subjected to internal and external factors such as hormones variations occurring in women’s life, phases of the menstrual cycle, drug treatments such as antibiotics and contraceptives, concomitant pathologies and life habits [1,2,3]. These factors can influence the fragile balance of the vaginal ecosystem, then creating a favorable environment to the development of opportunistic pathogens. The source of these pathogens can be both external (environment, sexual partner…) or internal, the gastro-intestinal tract being one reservoir of opportunistic pathogens able to colonize the vagina [4,5].

Therefore, vaginal infections occur when those pathogenic organisms develop, overgrow and induce inflammation associated with a range of symptoms potentially affecting women’s health and quality of life [6]. Among vaginal infections, bacterial vaginosis (BV) is mainly caused by the pathogen *Gardnerella vaginalis* with a prevalence estimated between 40% to 50% [6,7]. When the pathogen is a fungus, the corresponding infection is a vulvovaginal candidiasis (VVC) mainly caused by *Candida albicans* (in 90% of the cases) with an estimated prevalence of 20% to 35% [6]. Left untreated, these infections may cause severe complications of the upper genital tract (e.g., endometritis, salpingitis and pelvic inflammatory disease) leading to tubal scarring, infertility or ectopic pregnancies [8,9]. During pregnancy, these infections can increase the risk of preterm labor and low birth rate in the newborn [8,9]. Furthermore, some infections are associated with cellular abnormalities of the lower genital tract, which can lead to the development of cervical or vulvar dysplasia [9].

BV as well as VVC are difficult to diagnose and treat because the standard of care lies in the repeated use of “one-shot” antimicrobials, which normally consist of a cream and a vaginal device, available without medical prescription or microbiological determination of the infection. In the case of recurrences (defined as 4 infections or more per year), the treatment is based on the use of long-term oral antimicrobials. Considering the risk of developing multiple resistances to standard treatments, there is a real interest in alternative options for prophylactic and adjuvant-therapies [10]. The extensive use of antimicrobial treatments is also associated with a prolonged perturbation of the vaginal microbiota which cannot be spontaneously restored.

In this context, probiotics represent a promising alternative in preventing or restoring the balance of the vaginal microbiota [1]. Probiotics are defined as “live microorganisms that when administered in adequate amounts, confer a health benefit on the host” [11]. Few clinical studies evaluate the effects of probiotics on vaginal infections. Most of them tested a local administration, i.e., vaginal capsules, pessaries or tablets [12,13]. However, it seems relevant to also consider oral administration of the probiotics, which would constitute an easier and more convenient way, allowing a better compliance. The ability of the probiotic to migrate from the intestine to the vagina is essential for the efficacy of oral administration. This has already been described for probiotic bacteria, mainly *Lactobacilli* [14,15,16]. The oral intake of probiotics has been shown to exert an early action in the gastro-intestinal tract where pathogens of the genital area may constitute a reservoir (for example, *C. albicans)* [17].

The microbial community of the vagina plays an important role in the maintenance of a balanced ecosystem, thus preventing infections in women of reproductive age [1,2,3,4,5,6,7,8,9,10]. Most women present a vaginal microbiota typically dominated by few *lactobacilli* species, mainly *Lactobacillus crispatus*, *Lactobacillus jensenii*, *Lactobacillus iners* and *Lactobacillus gasseri* [1,18]. All of these species are a part of the Döderlein flora and play a protective role through different mechanisms such as the production of antimicrobial substances which inhibit pathogens’ growth [19,20,21]. The effects of a probiotic in maintaining this protective community may be evaluated when the probiotic is used to prevent vaginal infections.

The probiotic *Saccharomyces cerevisiae* CNCM I-3856 has already been evaluated in different experimental models of vaginal infections and has demonstrated strong antimicrobial properties against *C. albicans* and *G. vaginalis* [22,23,24] as well as a potential to decrease the inflammation induced by vaginal infections [22]. To our best knowledge, the migration of a (probiotic) yeast from the intestine to the vagina has never been described. This exploratory clinical study reveals new data on the behavior of the probiotic *Saccharomyces cerevisiae* CNCM I-3856, when consumed orally, including survival in the gastro-intestinal tract, recovery in vaginal samples and impact on the normal vaginal microbiota of healthy women.

## 2. Materials and Methods

### 2.1. Aim of the Study

The main objective of this study is to determine if the oral administration of the probiotic *Saccharomyces cerevisiae* CNCM I-3856 would lead to the detection of the strain in vaginal swab samples. The survival of the probiotic in the gastro-intestinal tract and its impact on the main bacterial species constituting the Döderlein flora are also assessed.

Our main hypothesis was that *S. cerevisiae* CNCM I-3856 was able to migrate from the intestine to the vagina after oral administration. In addition, we hypothesize that the oral administration route permits the conferment of probiotic benefits in both intestine and vagina, and the ease of administration for the women leads to increased compliance.

### 2.2. Population and Ethical Considerations

A total of 95 women were screened for this study. Among them, 60 fulfilled inclusion and non-exclusion criteria, 35 women were excluded because they did not meet inclusion criteria as presented in Table 1. Each participant was fully informed of study risks and benefits, aims and procedures. Prior to enrollment, each subject signed an informed consent form.

The study received the authorization from the French Health Authority (ANSM) and the ethics committee (CPP Nord Ouest III of Caen) under the registration number ID-RCB 2017-A02709-44 and was performed in compliance with the declaration of Helsinki and in accordance with the Good Clinical Practice (GCP) standards and the current French regulations. Moreover, the study was registered on www.ClinicalTrials.gov, under the unique identifier NCT 03574844.

### 2.3. Design

**Figure 1:**
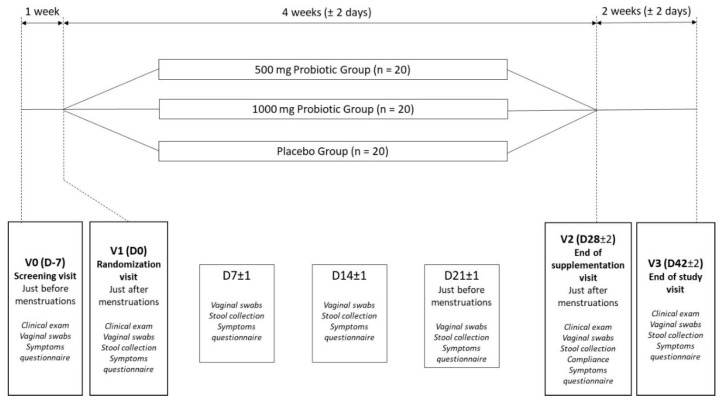
Experimental design of the clinical study (this diagram presents the study timeline (days, D) and the visits (V). For example, V1 corresponds to the randomization visit planned at D0 which is the first day of the supplementation phase).

This was an exploratory, randomised, double-blind, placebo-controlled, 3 parallel-groups clinical study. This monocentric protocol was run in a private investigation center (Biofortis Mérieux NutriSciences, Saint-Herblain, France) from March 2018 to January 2019. Sixty (60) healthy women were included and randomized into three different groups (*n* = 20 in each group), receiving one of the tested products or the placebo according to the randomization procedure and as depicted in Figure 1.

Menstruations are known to induce variations of the vaginal microbiota [25], hence the supplementation occurred between two menstrual periods for the duration of a menstrual cycle (28 days +/−2 days). The first visit (V0) was a screening visit scheduled just before the menstrual periods of potential subjects. The women were assessed based on the inclusion and non-inclusion criteria as presented in Table 1. Eligible women performed 2 self-collected vaginal swabs and were asked to provide a stool sample before the next visit. The vaginal swabs were used to confirm the healthy status of the vaginal microbiota of subjects using the Nugent score, the analysis of specific pathogens such as *Trichomonas* and *Candida* spp. and the determination of the vaginal pH. The screening visit was followed by a randomization visit (V1) planned for no later than 1 week after the end of the menstrual period. The randomization occurred as soon as the healthy status of the vaginal microbiota was confirmed. Two self-collected vaginal swabs and stool collection were planned. The randomized subjects started the supplementation phase for 28 days (+/−2 days), consuming once daily one of the tested products or the placebo (from V1 to V2). Vaginal swabs and stool samples were collected at days 7, 14, 21, 28 (±2 days) to perform the detection and quantification of *S. cerevisiae* CNCM I-3856 and *C. albicans*. The two last swabs were performed just before the next menstrual period. At the end of the supplementation phase (V2), a follow-up phase started for 14 days in order to assess the presence of the probiotic in samples at 42 days ± 2 days (V3).

All along the intervention, drug treatments, medical devices and dietary supplements potentially having an impact on the vaginal microbiota were prohibited. As mentioned in Table 1, the treatments concerned included chronic corticosteroids, chronic immune modulators (immunostimulants, immunosuppressants…), antibiotics, antifungals, probiotics, prebiotics, symbiotics, phytoestrogens and local estrogens. These instructions were given at the screening visit (V0) to each participant. The potential consumption of prohibited products was monitored all along the study and registered in case report forms (CRF) at each visit.

### 2.4. Intervention

The study evaluated a probiotic dietary supplement provided by Gnosis by Lesaffre (a Business Unit of Lesaffre Group, France). This dietary supplement is 100% composed of *Saccharomyces cerevisiae* CNCM I-3856. This strain is a proprietary and patented strain of Lesaffre, registered in the French National Collection of Microorganisms (CNCM). *S. cerevisiae* species was determined using phenotypic (API^®^ID32C, Biomerieux SAS, Marcy l’Etoile, France) and genotypic methods (genetic amplification and sequencing of 26S DNA) [26,27]. Moreover, the strain I-3856 has been characterized by polymerase chain reaction (PCR) Interdelta typing techniques [28] and complete genome sequencing. In this clinical study, two different doses of the probiotic were tested:

- 2.5 × 10^9^ colony forming units (CFU) daily in the 500 mg Probiotic group;
- 5 × 10^9^ CFU daily in the 1000 mg Probiotic group.

The comparative product was a placebo (Placebo group) composed of inactive ingredients i.e., maize starch and magnesium stearate. All capsules were presented in blisters and manufactured by PILEJE Industries, France. They were hydroxypropyl methylcellulose (HPMC) capsules of same size, color and taste whatever the group and were prepared according to good manufacturing practices. For practical reasons, two types of capsules were manufactured: the first type contained the probiotic at 2.5 billion CFU and the second type contained maize starch and magnesium stearate. Different combinations of the two types of capsules allowed to supply each of the study groups according to Table 2. Every subject consumed two capsules per day, just before breakfast with a glass of water.

### 2.5. Assesment

#### 2.5.1. Study Endpoints

The primary endpoint of the study was the proportion of women with *S. cerevisiae* CNCM I-3856 in vaginal samples, detected using specific microbiological methods (subcultures in two culture media followed by molecular typing to confirm the presence of I-3856) after a 4-week supplementation period. Secondary endpoints were the proportion of women presenting the strain in vaginal samples at days 7, 14 or 21 after the beginning of the supplementation and at day 42 which corresponded to the end of the follow-up phase. The presence and survival of the probiotic were assessed respectively through the detection of the strain and quantification of CFU in stool and vaginal samples, by serial dilutions. The impact of the probiotic on the Döderlein flora was measured through the quantification and determination of the changes from baseline for *Lactobacillus crispatus*, *Lactobacillus iners*, *Lactobacillus gasseri* and *Lactobacillus jensenii* using a quantitative PCR (qPCR) method. The presence of the probiotic yeast in the vaginal microbiota considering its presence in intestinal microbiota has been studied. Moreover, the correlation between the quantity of the strain retrieved in intestinal and vaginal microbiota was assessed. Finally, the presence and the changes from baseline of the pathogen *C. albicans* in vaginal samples was investigated at each study time point by subculture steps and quantification of CFU.

#### 2.5.2. Laboratory Assessments

At the screening visit, two self-collected vaginal swabs were performed to confirm the normal status of the vaginal microbiota of each subject. The first swab was used for vaginal pH determination (normal vaginal pH ≤5) using auto-test DOSATEST^®^ (VWR chemicals, Leuven, Belgium). The other swab was used both for the determination of the Nugent score (vaginal flora is considered as normal when the Nugent score is ≤3) by coloring with the Gram technique and for the detection of specific pathogens such as *Candida albicans* and *Trichomonas* using agar plate SGC^®^ (bioMérieux, Craponne, France).

After randomization, vaginal samples were collected using two different swabs. The first was a dry swab (FLOQSwabs^®^, Copan, Carlsbad, California, USA) used for the detection and the quantification of the 4 main species composing the Döderlein flora, the determination of the Nugent score and the detection of specific pathogens (i.e., *Candida albicans*, *Trichomonas*). The second one was a nylon flocked swab (eSwab liquid Amies, Copan) used for the detection and the quantification of the strain I-3856 and *Candida albicans*. Subjects were asked to avoid sexual intercourse 24 h prior to each vaginal swab collection. Subjects were not allowed to use any intravaginal products (e.g., spermicides, lubricants etc.) 24 h prior to sample collection. Following direct vaginal sample plating on ChromID Candida CAN2 (bioMérieux, Lyon, France), swabs were then stored at −80 °C.

After randomization (V1), the impact of the tested probiotic on the main bacteria species constituting the Döderlein flora (*L. crispatus*, *L. gasseri*, *L. iners*, *L. jensenii*) was assessed at each study timepoint. Total bacterial DNA were extracted from vaginal swabs (FLOQSwabs^®^, Copan) using the phenol-chloroform method. Changes in the populations of the four bacteria studied were assessed using qPCR (ABI 7500 Real Time PCR System) [29].

Subjects were asked to perform a stool collection no later than 36 h prior to each visit. All required materials (pots, cooler, ice pack and operating procedures) were provided to the subjects. Collection pots were kept between +2 °C and +8 °C before bringing them back to the Clinical Investigation Center. Aliquots of 5 g of fresh stool were then immediately plated on agar plates.

For both vaginal and fecal samples, dilutions were performed in order to estimate the quantity of *C. albicans* and *S. cerevisiae*. Stool samples (aliquots of 5 g) and vaginal swabs (eSwab liquid Amies, Copan) were firstly plated on ChromID Candida CAN2 agar plates (bioMérieux, Lyon, France) and incubated for 48 to 72 h at 30 °C. This first step allowed the detection of *C. albicans* as blue colonies. The two yeasts species were distinguished according to the macroscopic observation of colonies. White colonies were then subcultured on BBL™ CHROMagar™ Candida Medium (BD, Heidelberg, Germany) for 48 to 72 h at 30 °C in order to select *S. cerevisiae*. This second step allowed the selection of purple colonies before processing to Inter delta typing using the PCR technique [30] for the identification of presumptive I-3856 clones. In that aim, colonies were subcultured on yeast malt (YM) medium and genomic DNA were extracted using InstaGene™ Matrix kit (Bio-Rad, Marnes-la-Coquette, France). PCR typing of I-3856 strain was performed and capillary electrophoresis (Fragment Analyser, Agilent, Santa Clara, California, USA) was used for the migration of amplicons. Inter delta profiles obtained were compared with the positive control I-3856. This final step of molecular typing allowed to detect the presence of the probiotic strain I-3856 and to estimate the number of CFU/sample in vaginal swabs and stool samples. The limit of detection for *C. albicans* and *S. cerevisiae* in the vaginal content was of 10 CFU/sample and in the intestinal content of 100 CFU/sample. In subsequent quantitative analyses, the counts of the probiotic strain I-3856 in stool samples were normalized to the quantity of stool (CFU per gram of stool); this was not possible for vaginal swabs and quantities were expressed as CFU per vaginal swab.

#### 2.5.3. Safety and Compliance Assessment

The safety criteria were based on the descriptive statistics of the number of symptoms of vaginal infection assessed by the investigator with a specific symptoms questionnaire at each study time-point (D-7 to D42); and on adverse events occurring all along the study (from V0 to V3).

Compliance was determined through the assessment of returned packaging and interview of the subjects at each visit.

#### 2.5.4. Sample Size, Randomization Procedure and Statistical Analysis

Twenty (20) subjects per arm were included in the present study (active product n°1/active product n°2/placebo), considering an anticipated low dropout rate usually observed in this type of study. Consequently, 60 subjects were included. The number of subjects to include was determined following guidelines on sample size for pilot study [31,32].

After the screening phase, each subject was assigned to one of the three groups (active product n°1, active product n°2 or placebo). The product allocation list was generated using SAS^®^ software (version 9.3 or higher, SAS Institute Inc., Cary, NJ, USA) before the study start-up. Allocation of product depended only on the subjects’ inclusion sequence. The investigator allocated the product in accordance with the randomization number assigned to the subject during the inclusion visit (V1). Statistical analyses were performed using SAS^®^ software version 9.4 (SAS Institute Inc., Cary, NC, USA). For all statistical tests, the 0.05 level of significance was used to justify a claim of a statistically significant effect.

To study the impact of the probiotic on the Döderlein flora, we investigated the changes D7–D0; D14–D0; D21–D0; D28–D0; D42–D0 and D42–D28 of the following four dominant *Lactobacillus* species of the Döderlein flora: *Lactobacillus crispatus*, *Lactobacillus gasseri Lactobacillus jensenii* and *Lactobacillus iners.*

The count of *Lactobacillus* at each day from D0 to D42 was modeled using the following mixed effect model for repeated measurements:

$$Y_{Dn}=Product+Day+Product*Day+\varepsilon$$

with:

- $Y_{Dn}$: Value of the parameter at Day *Dn;*
- Product: Placebo, 500 mg or 1000 mg of *S. cerevisiae* CNCM I-3856;
- Day *Dn* (D0 D7 D14 D21 D28 or D 42);
- ε Residuals with covariance structure considering the repeated measurements on the same subject.

The presence of *S. cerevisiae* CNCM I 3856 in the vaginal samples considering its presence in the intestinal sample was studied for each active group (placebo excluded) using the following marginal correlated logistic model:

(1) $$log\left[\frac{P\left(Y_{Dn,\;Sph}=1\right)}{P\left(Y_{Dn,\;Sph}=0\right)}\right]=Dose+Day+Sphere+\varepsilon$$

with:

- $Y_{Dn,Sph}$: 1 if the CNCM I 3856 at day *Dn* in the sphere *Sph* is present, 0 if absent;
- Dose: 500 mg or 1000 mg of *S. cerevisiae* CNCM I-3856;
- Day: Day of the study (D7, D14, D21, D28, D42);
- Sphere: Anatomical Sphere *Sph* (vaginal or intestinal).

The interactions (2-way and 3-way interactions) have been considered and assessed but were removed because of non-convergence of the model.

## 3. Results

### 3.1. Study Flowchart and Baseline Characteristics of the Population

A total of 60 women were included in this study and randomly allocated to one of the three groups (*n* = 20 in each group). Baseline characteristics of the population are presented in Table 3. The quantitative variables are expressed as mean (standard deviation, SD) and qualitative results as n (%). All groups were similar regarding demographic characteristics prior to enrollment in the study protocol.

**Figure 2:**
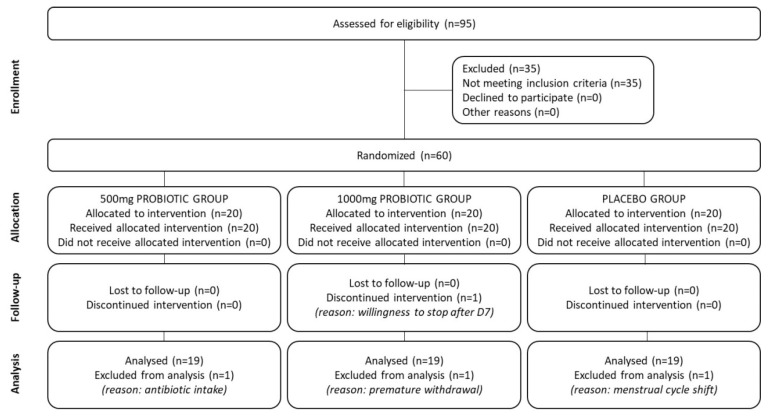
Clinical study diagram according to Consolidated Standards of Reporting Trials (CONSORT) guidelines.

Fifty-seven (*n* = 57) women completed the study. Three (*n* = 3) women did not (protocol deviation (*n* = 2), consent withdrawal (*n* = 1)). Thus, fifty-seven (*n* = 57) women were included in the efficacy analysis population (Per Protocol) (500 mg Probiotic group: *n* = 19; 1000 mg Probiotic group: *n* = 19; Placebo group: *n* = 19). The flow chart of the study is presented in Figure 2.

Compliance was close to 99%.

### 3.2. The Migration of *S. cerevisiae* CNCM I-3856 from Intestine to Vagina Was Demonstrated

Over the 4-week supplementation phase, *S. cerevisiae* CNCM I-3856 has been detected in the vaginal samples of 21% of women (*n* = 4/19) in the 500 mg Probiotic group and 16% of women (*n* = 3/19) in the 1000 mg Probiotic group. 

**Figure 3:**
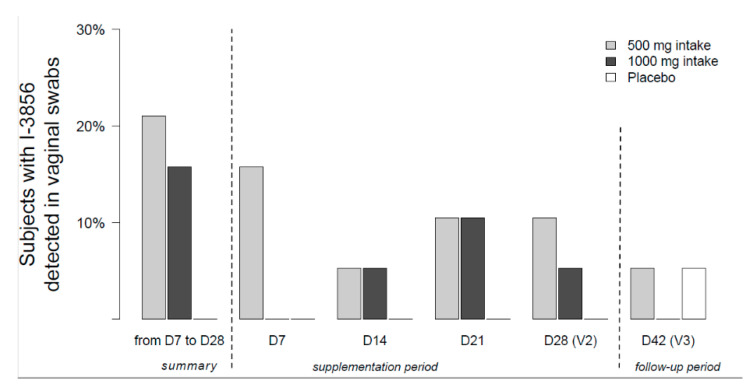
Proportion of women (%) with *S. cerevisiae* CNCM I-3856 detected in vaginal samples at each study timepoint (*n* = 19 for Probiotic 500 mg group, *n* = 19 for Probiotic 1000 mg group and *n* = 19 for placebo group).

The proportion of women presenting the probiotic in vaginal samples at each study timepoint is depicted in Figure 3:

- 500 mg Probiotic group (*n* = 19): 15.8% at day 7, 5.3% at day 14, 10.5% at day 21 and 10.5% at day 28.
- 1000 mg Probiotic group (*n* = 19): 0% at day 7, 5.3% at day 14, 10.5% at day 21 and 5.3% at day 28.

At the end of the follow-up phase (day 42), the probiotic has been detected in one woman (*n* = 1/19) included in the 500 mg Probiotic group.

Moreover, the results confirmed there is more chance to detect the strain I-3856 in stool samples than in vaginal samples (odds ratio (OR) = 0.07 [confidence interval (CI) 95%: 0.02; 0.19]; *p*-value < 0.0001, marginal correlated logistic model excluding the placebo group without interaction terms).

An estimated quantity of 1 to 5 log/swab of *S. cerevisiae* CNCM I-3856 is recovered as detailed in Table 4.

The study established a weak correlation between the quantity of probiotic retrieved in stool samples and vaginal samples (Spearman’s rank correlation coefficient <0.40).

### 3.3. No Significant Impact of *S. cerevisiae* CNCM I-3856 on the Döderlein Flora Was Found

All along the study, no statistically significant differences between groups were found regarding the variations of the populations of *L. crispatus*, *L. iners* and *L. jensenii.* The population of *L. gasseri* showed a significant increase on the variation D14-D0 in the 500 mg Probiotic group as compared to the Placebo group (1.88 [0.74; 3.02] CI 95%, *p* = 0.002, mix model considering product intake and time with interaction).

### 3.4. *S. cerevisiae* CNCM I-3856 Survived through the Gastrointestinal Tract

Over the supplementation phase, *S. cerevisiae* CNCM I-3856 has been detected in stool samples in 80% of women (*n* = 15/19) included in the 500 mg Probiotic group and 100% of women (*n* = 19/19) included in the 1000 mg Probiotic group. The probiotic was not detected in the stool samples of women included in the Placebo group. At the end of the follow-up phase (day 42), no women presented the strain in stool samples.

**Figure 4:**
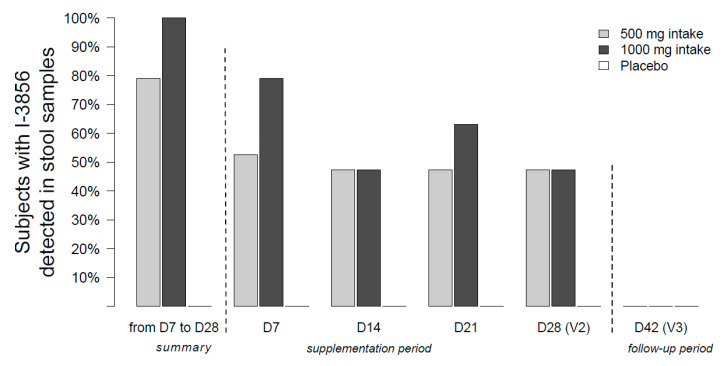
Proportion of women (%) with *S. cerevisiae* CNCM I-3856 detected in stool samples at each study timepoints (*n* = 19 for Probiotic 500 mg group, *n* = 19 for 1000 mg group and *n* = 19 for placebo group).

The proportion of women presenting the probiotic in stool samples at each study timepoints is described in Figure 4.

**Figure 5:**
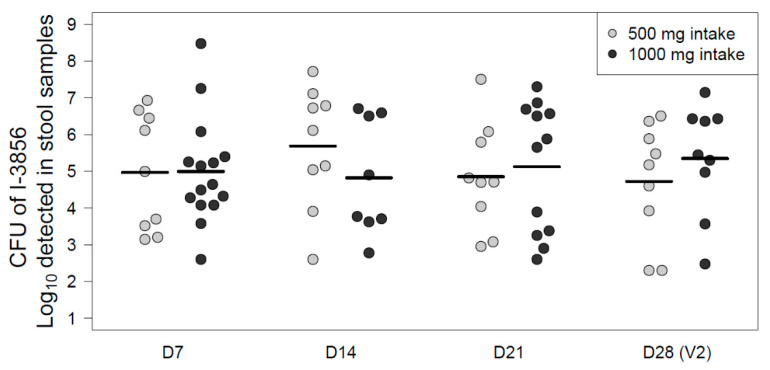
Estimated CFU counts of *S. cerevisiae* CNCM I-3856 detected in stool samples at each study timepoint expressed as log10 CFU/sample. Only the samples in which the yeast was detected are figured in this plot.

As presented in Figure 5, the average quantity of *S. cerevisiae* CNCM I-3856 recovered from stool samples over the supplementation phase (D7-D28) was 5.8 log CFU/g in both groups receiving the probiotic.

### 3.5. *C. albicans* Did Not Show Significant Variations in Vaginal Samples

No significant differences were shown between groups for the detection and quantification of *C. albicans* in vaginal samples (Table 5).

### 3.6. *S. cerevisiae* CNCM I-3856 Was Safe and Well Tolerated

No serious adverse event linked to the research nor the tested products was recorded whatever the study group. The tolerance of the tested product was excellent.

## 4. Discussion

For the first time, this exploratory clinical study allowed to demonstrate the ability of the probiotic *S. cerevisiae* CNCM I-3856 to migrate from the intestine to the vagina in healthy women. Over the supplementation phase, the detection of the probiotic strain in vaginal swabs was observed in 21% (*n* = 4/19) of women included in the 500 mg Probiotic group and 16% (*n* = 3/19) of women included in the 1000 mg Probiotic group.

One of the most critical points of this protocol was the implementation of the collection and analysis of vaginal swabs. Although the technique of self-sampling has been validated in various studies [33,34] and clear instructions were given to the participants, results from self-collected samples remained difficult to interpret for several reasons. First, the exact quantity of biological material collected using swabs remains unknown contrary to what can be measured with feces. Large variations between vaginal swabs are possible and can lead to differences in the CFU counts. At the stage of collection, the delay between self-sampling and analysis could also lead to underestimation of the probiotic CFU counts. At the stage of the analysis, the implemented method involving culture before molecular typing by PCR added to the stringency of the protocol. As such, the limits of our analytical protocol could have likely led to an underestimation of the proportion of women presenting a confirmed probiotic migration. Indeed, although positive samples were 100% real-positive, owing to a very specific molecular typing technique that detects cultivable and metabolically active yeast cells, negative samples may contain viable but not cultivable cells, resulting in false negatives. Consequently, the presence of the probiotic in more samples, but in a concentration below the limit of detection of the method, cannot be excluded. Although we may have underestimated the migration of I-3856, our results are comparable to previous data obtained with probiotic bacteria in clinical studies using comparable microbiological methods. Thus, in the study of Bohbot et al., the probiotic strain *Lactobacillus casei rhamnosus* (LCR35) was detected in 25% of women [14]. Several other clinical studies were previously conducted to assess the transfer of probiotic bacteria from the intestine to the vagina after oral administration; vaginal detections reaching between 0% and 80% of women [14,15,16,33,34,35,36,37,38,39]. In some of these studies, the tested product was a blend of two or three different probiotic strains, the migration being considered as confirmed when at least one of the strains was detected at a certain timepoint which may result in overestimating the proportion of positive samples to the presence of a particular strain [39]. Moreover in some of these studies, the PCR-based detection methods for the probiotic were only specific to species (and not to the strain) [33,34,37,40]. These methodological limits may induce overestimation of the detection of probiotics in the vaginal samples.

The choice of the microbiological method would be crucial since the use of qPCR without culturing step may detect not only viable cells but also dead cells from the tested probiotic [40,41,42]. In this study, the presence of dead I-3856 cells in stool or vaginal samples was not studied but we already described the properties of inactivated I-3856 against *C. albicans* and its capacity to induce the pathogen clearance in experimental models of vulvovaginal candidiasis [23]. For further investigation, the potential role of dead cells would gain to be considered using direct PCR.

Considering our current approach as semi-quantitative, the quantity of *S. cerevisiae* CNCM I-3856 retrieved in vaginal samples is estimated to vary between 1 to 5 log CFU/sample over the supplementation period in both probiotic groups. No significant difference was found between the groups, neither for the detection nor the quantification of the strain. The probiotic dosages were very close between the groups (2.5 and 5 billion CFU/g), probably explaining the absence of dose-effect. A recent metanalysis discussing the dose-responses of probiotics in human studies concluded there is no homogenous picture on the influence of the dose on probiotics efficacy and more dedicated dose-response studies are needed with less heterogeneity [41]. For the rest of the discussion henceforth, we suggest considering both probiotic groups as one.

Another important finding of this study is the *a priori* absence of negative impact of *S. cerevisiae* CNCM I-3856 on the four main *lactobacilli* constituting a normal vaginal microbiota. Using qPCR, we were not able to find significant variations in the populations of *L. crispatus*, *L. jensenii* and *L. iners* when comparing the groups over the supplementation period (data not shown). *L. gasseri* population was significantly increased on the period D0–D14 in one of the probiotic groups only. Reported as a dominant species of *lactobacilli* in the vagina of healthy women, the presence of *L. gasseri* is described to be negatively correlated with vaginal disorders such as BV and may confer resistance to the colonization by pathogens through competitive adhesion and direct inhibition involving lactic acid, hydrogen peroxide and bacteriocins production [42].

In this study, we also demonstrated the ability of the strain CNCM I-3856 to survive in the gastro-intestinal environment after oral administration. Over the supplementation phase (D0–D28), the strain was detected in the feces of high proportions of women consuming the probiotic; namely 90% (*n* = 34/38) (Figure 3). Moreover, averaged survival counts were of 5.9 log CFU per gram of feces in both groups confirming the high survival of the strain after its passage through the gastro-intestinal tract (Figure 4). Previous studies described comparable results with another yeast (*S. cerevisiae* var. *boulardii),* the recovery rate in feces being between 5 to 7 log per gram [43,44,45]. Referring back to *S. cerevisiae* CNCM I-3856, Cordonnier et al. described the survival of the strain to gastric acidity using a dynamic in vitro model of human digestive tract. According to these results, as much as 6.4 log_10_ ± 0.05 CFU/mL of cultivable yeast cells were likely to reach the human colon where they are supposed to exert their benefits on the host [46].

The fact that the tested probiotic can survive through the gastrointestinal tract could promote an additional beneficial effect to its potential action in the vagina. Indeed, the gastro-intestinal tract was identified as a potential source of contamination of the genital tract. Physical proximity between both systems allows interactions in-between microbial communities and migration of pathogens such as *C. albicans* [10,36,47,48]. Thus, there is an interest in administering probiotics orally allowing antipathogenic activities from the intestine and then directly in the vagina.

The compliance observed in this trial is particularly high; ~99% for all population. We assumed the oral route would be easy and convenient for a long use, increasing the compliance and allowing a benefit of the probiotic in the intestine and the vagina. This strongly contrasts with most of the products available on the women’s health market, since the main administration route is the local (vaginal) one for a direct action on site. *S. cerevisiae* CNCM I-3856 confirmed its excellent tolerability, as already observed in several clinical studies [49]. In this clinical study, it is interesting to note that neither the presence nor the quantity of the pathogen *C. albicans* in vaginal samples changed significantly throughout the study. Although this pathogen was not detected in any women at the inclusion stage, *C. albicans* was detected in few samples at some study time points (10 women out of 60~6%). However, these were not related to specific adverse events such as candidiasis. It confirmed healthy carriage in 10% to 15% of asymptomatic women colonized with *Candida* species [50,51]. Moreover, *C. albicans* was never detected in vaginal samples when the probiotic was detected and conversely, the probiotic was never detected in samples positive for *C. albicans*. These results may support previous studies showing the ability of *S. cerevisiae* CNCM I-3856 to positively influence the course of vaginal infections by accelerating the clearance of pathogens due to multiple interactions of *S. cerevisiae* with *C. albicans* and *G. vaginalis* [22,23,24,52]. Interactions between *S. cerevisiae* and *C. albicans* include promotion of coaggregation, inhibition of adherence to epithelial cells, and inhibition of *Candida* virulence factors [23].

Additional future investigations could include a large-scale validation of the clinical efficacy of *S. cerevisiae* CNCM I-3856 for the prevention of recurrent vulvo-vaginal candidiasis. Considering also that the ability of a probiotic to migrate from intestinal to vaginal sphere is probably multifactorial and not only associated with the strain properties, the implementation of a new protocol would be the opportunity to study the influence of inter-individual morphology and genetic background, intrinsic factors such as age, smoking, ethnic groups and environmental factors (i.e., douching, sexual activity, hormonal changes periods…) on the vaginal microbiota composition [53] and the migration of a probiotic after oral administration.

## 5. Conclusions

This exploratory clinical study demonstrates the recovery of *S. cerevisiae* CNCM I-3856 in vaginal samples after oral administration. The probiotic showed a high survival rate through its passage in the gastrointestinal tract, then migrating to the vagina without impacting the Döderlein flora. Although the stringency of the microbiological method implemented may have underestimated the migration, a daily oral dose of 2.5 billion CFU of *S. cerevisiae* CNCM I-3856 allowed the detection of the strain in vaginal samples with a high level of reliability and safety. Oral use of the probiotic rather than local administration certainly led to an increased compliance and an early antipathogenic action of the probiotic into the intestine, which is known to be one of the potential sources of vaginal infection. Once in the vagina, the probiotic may also exert its benefits locally. Although larger clinical studies are needed, the potential of *S. cerevisiae* CNCM I-3856 as an alternative option for the management of vaginal infections is worthy of consideration.

## Figures and Tables

**Table 1:** Inclusion and Exclusion Criteria as Defined in the Study Protocol.

Inclusion Criteria	Exclusion Criteria
Caucasian or Asian women, With regular menstrual cycles, assessed as 28 days ± 3 days, With a normal vaginal flora defined as: ∘ Nugent score ≤3, ∘ Vaginal pH ≤5, ∘ Absence of *C. albicans*, *Trichomonas,* Effective contraception method without any changes for the duration of the study, No changes in daily routine for the duration of the study (hygiene, dietary habits, tobacco and alcohol consumption, physical activity and sexual life), Good general and mental health compatible with the study participation, Tobacco consumption ≤10 cigarettes per day, Able and willing to participate to the study by complying with the protocol procedures, Subject affiliated to a health social security system.	Ongoing symptoms of vaginal and/or urinary infection at V0 and V1 visits, History of vulvo-vaginal pathological conditions, Currently pregnant, lactating, or intending to be pregnant, Intolerance to any of the study products, Suffering from a metabolic disorder or severe chronic disease, Recent history of radiotherapy, Menopausal or pre-menopausal, With menstruation periods usually lasting more than 7 days, Under chronic treatment with corticosteroids and/or immune modulator or medication or dietary supplement, oral or local, which could affect study parameters, Unable to communicate with the investigator.

**Table 2:** Study products.

Products	*S. cerevisiae* CNCM I-3856	Maize Starch + Magnesium Stearate	Total
500 mg Probiotic Group	1 capsule (500 mg) 2.5 × 10^9^ CFU	1 capsule (500 mg)	2 capsules (2 × 500 mg)
1000 mg Probiotic Group	2 capsules (2 × 500 mg) 5 × 10^9^ CFU	-	2 capsules (2 × 500 mg)
Placebo Group	-	2 capsules (2 × 500 mg)	2 capsules (2 × 500 mg)

CFU: Colony Forming Units.

**Table 3:** Baseline characteristics of the study population.

	Total Included Subjects (*n* = 60)	Placebo Group (*n* = 20)	500 mg Probiotic Group (*n* = 20)	1000 mg Probiotic Group (*n* = 20)
**Age** (years)	31.2 (7.19)	32.2 (7.67)	32.7 (7.57)	28.7 (5.83)
**Body mass index (BMI)** (Kg/m^2^) (V1)	23.8 (4.90)	23.5 (4.16)	24.6 (6.22)	23.3 (4.19)
**Ethnicity**	60 (100%)	20 (100%)	20 (100%)	20 (100.0)
Caucasian	60 (100%)	20 (100%)	20 (100%)	20 (100.0)
**Contraceptive method**	60 (100%)	20 (100%)	20 (100%)	20 (100.0)
Estroprogestative pill (21 days/month)	38 (63.3%)	13 (65.0%)	13 (65.0%)	12 (60.0%)
Simple intra-uterine device	19 (31.7%)	5 (25.0%)	7 (35.0%)	7 (35.0%)
Oestroprogestative patch with a stop of 7 days at each cycle	1 (1.7%)	0 (0.0%)	0 (0.0%)	1 (5.0%)
Tubal ligation	1 (1.7%)	1 (5.0%)	0 (0.0%)	0 (0.0%)
Essure system	0 (0.0%)	0 (0.0%)	0 (0.0%)	0 (0.0%)
Condom + spermicidal gel	1 (1.7%)	1 (5.0%)	0 (0.0%)	0 (0.0%)
**Vaginal pH** (V0)	4.10 (0.303)	4.15 (0.366)	4.03 (0.112)	4.13 (0.358)
**Nugent score** (V0)	0.52 (0.676)	0.40 (0.681)	0.4 (0.598)	0.75 (0.716)
**Presence of *C. albicans* in vaginal samples** (V1)	2 (3.3)	1 (5.0)	0 (0.0)	1 (5.0)
**Presence of *S. cerevisiae* I-3856 in stool samples** (V1)	0 (0.0)	0 (0.0)	0 (0.0)	0 (0.0)
**Presence of *S. cerevisiae* I-3856 in vaginal samples** (V1)	0 (0.0)	0 (0.0)	0 (0.0)	0 (0.0)

Values are presented as mean (standard deviation, SD) or number (percent).

**Table 4:** Quantification of *S. cerevisiae* CNCM I-3856 in the vaginal samples positive to the detection of the strain at each study timepoint, expressed as log/sample.

Timepoints	500 mg Probiotic Group		1000 mg Probiotic Group	
	Randomization Number	Log/Sample	Randomization Number	Log/Sample
**D7**	26	1.0	-	-
	60	2.0	-	-
	67	1.8	-	-
**D14**	67	3.9	84	5.0
**D21**	4	1.6	41	4.5
	67	4.5	84	1.0
**D28**	4	1.0	74	1.3
	67	4.9	-	-

**Table 5:** Quantification of *C. albicans* in the vaginal samples where it was detected, at each study timepoint, expressed as log/sample.

Timepoints	500 mg Probiotic Group		1000 mg Probiotic Group		Placebo Group	
	Randomization Number	Log/sample	Randomization Number	Log/Sample	Randomization Number	Log/Sample
**D0**	-	-	17	2.4	45	4.2
**D7**	-	-	17	3.5	05	2.2
	-	-	-	-	43	3.0
	-	-	-	-	45	2.7
**D14**	01	1.0	17	2.8	05	3.3
	-	-	-	-	43	4.5
**D21**	32	3.3	17	3.7	39	1.0
	-	-	-	-	43	3.6
	-	-	-	-	45	3.0
**D28**	70	2.1	17	2.0	05	1.9
	-	-	-	-	45	1.6
